# Social-emotional need satisfaction, prosocial motivation, and students’ positive behavioral and well-being outcomes

**DOI:** 10.1007/s11218-022-09691-w

**Published:** 2022-04-19

**Authors:** Rebecca J. Collie

**Affiliations:** School of Education, University of New South Wales, Sydney, NSW 2052 Australia

**Keywords:** Social and emotional competence, Prosocial motivation, Emotional well-being, Basic psychological need satisfaction

## Abstract

This study investigated the extent to which students’ social-emotional basic psychological need satisfaction (of social-emotional autonomy, social competence, relatedness with students, and relatedness with teachers) is associated with their prosocial motivation and, in turn, behavioral and emotional well-being outcomes. For motivation, autonomous prosocial motivation and controlled prosocial motivation (by way of introjected and external regulation) were examined. Behavioral outcomes comprised prosocial behavior and conduct problems reported by parents/carers, and emotional well-being were positive and negative affect reported by students. With data from 408 secondary school students (and their parents/carers), structural equation modeling showed that perceived autonomy was associated with lower negative affect. Perceived social competence was associated with greater autonomous prosocial motivation, lower external prosocial regulation, greater positive affect, and lower negative affect. Relatedness with teachers was associated with greater autonomous prosocial motivation. In turn, autonomous prosocial motivation was associated with greater prosocial behavior, whereas external prosocial regulation was associated with lower prosocial behavior. Introjected prosocial regulation was only associated with greater negative affect. The results hold implications for promoting social and emotional competence among students.

**Supplementary Information:**

The online version contains supplementary material available at 10.1007/s11218-022-09691-w.

## Introduction

Social and emotional competence (SEC) is crucial for helping humans to navigate their interactions and experiences in the world (Domitrovich et al., 2017). SEC refers to “effective management of intrapersonal and interpersonal social and emotional experiences in ways that foster one’s own and others’ thriving” (Collie, 2020, p.77). In the past, SEC has largely been examined by focusing on its outward manifestations, such as via the capacities (e.g., relationship skills) or behaviors (e.g., cooperative behavior) that individuals enact (Stump et al., 2009). This prior research has provided important understanding about SEC and has also informed a robust literature on how SEC can be promoted via social and emotional learning programs (Domitrovich et al., 2017; Weissberg et al., 2015). Recently, however, researchers have highlighted the importance of also considering factors that underpin SEC—that is, underlying mechanisms comprising perceptions and motivation (Stump et al., 2009). Mechanisms are critical to consider given they determine the manner and extent to which an individual enacts manifestations. For instance, although a student may possess the capacity or know the behavior required to respond constructively in class discussions (a manifestation of SEC), the extent to which they actually do so is impacted by underlying mechanisms of SEC (Collie, 2020; Rose-Krasnor & Denham, 2009). Researchers have, therefore, called for studies that simultaneously investigate both the mechanisms and manifestations of SEC. This is essential for advancing knowledge of what factors can be addressed in interventions to promote enactment of socially and emotionally competent behaviors. Although research examining both mechanisms and manifestations is emerging, notable gaps remain. In particular, prior research has yet to consider the two key underlying mechanisms (perceptions and motivation) simultaneously. Doing so is necessary to provide a more complete picture about how to better foster SEC among students.

**Fig. 1:**
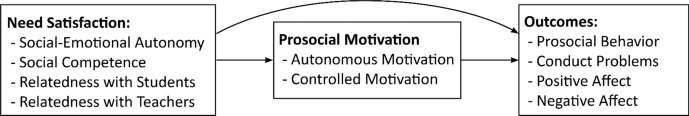
Hypothesized model

The aim of the present study was to examine the role of both mechanisms—perceptions and motivation—in relation to important behavioral and well-being outcomes among a sample of secondary school students. Secondary school is a time where students are engaged in increasingly complex social and emotional interactions (Eccles et al., 1993). It is also a time when the importance of peer approval and motivations for conforming to peer norms become heightened (Tomova et al., 2021), which can lead to declines in prosocial behavior (Padilla-Walker et al., 2018). Thus, examining mechanisms that might be implicated in students’ enactment of prosocial behaviors, as well as their well-being outcomes, is important for building understanding and guiding intervention development. The perceptions examined in the current study were operationalized by way of social-emotional basic psychological need satisfaction: perceived autonomy, competence, and relatedness. Prosocial motivation was examined as the motivational mechanism. More specifically, autonomous (i.e., intrinsic and identified regulation) and controlled (i.e., introjected and external regulation) prosocial motivation were investigated. The manifestations were (parent/carer-reported) prosocial behavior and conduct problems, along with (student-reported) emotional well-being (i.e., positive and negative affect). As Fig. 1 shows, the hypothesized model positions the perceptions as predictors of the prosocial motivation factors and, in turn, the behavioral and emotional well-being outcomes (while controlling for background characteristics).

### Conceptual framework

The Social and Emotional Competence (SEC) School Model (Collie, 2020) acted as the guiding conceptual framework for the present study. Grounded in motivational theory (e.g., self-determination theory; Ryan & Deci, 2017) and conceptualizing from the SEC literature (e.g., Rose-Krasnor & Denham, 2009), the SEC School Model highlights the mechanisms and manifestations inherent in SEC and its development. More precisely, the SEC School Model aims to inform understanding of how socially and emotionally competent behaviors can be fostered by shedding light on the mechanisms that underlie them. In addition, the model also highlights the role of mechanisms in supporting well-being outcomes (Collie, 2021). Two mechanisms are posited in the SEC School Model: perceptions (by way of social-emotional basic psychological need satisfaction) and social-emotional motivation (by way of autonomous vs. controlled motivation). Motivation theories have long highlighted the importance of perceptions as underpinning motivation (e.g., Bandura, 1997; Ryan & Deci, 2017). The SEC School Model adopts this understanding and extrapolates it to the social and emotional domains.

The central process in the SEC School Model establishes that social-emotional need satisfaction (i.e., autonomy, competence, and relatedness) predicts social-emotional autonomous motivation and, in turn, adaptive behavioral and well-being outcomes. The present study involved an examination of this central process, where motivation was operationalized by way of prosocial motivation. Prosocial motivation was selected because it is a well-recognized type of social-emotional motivation and because it has previously been shown to be important for a range of positive outcomes (e.g., Roth et al., 2011). The present study extends prior research by considering social-emotional need satisfaction as a predictor of autonomous prosocial motivation—thus, providing knowledge about both types of mechanisms and their interrelations. Alongside autonomous motivation, this study also considered the role of controlled prosocial motivation. Each factor in the study is introduced below, beginning with social-emotional need satisfaction.

### Social-emotional need satisfaction as a foundational mechanism of SEC

For decades, motivation researchers have highlighted the importance of three basic psychological needs for boosting motivation: *perceived autonomy* refers to the sense of being the origin of one’ behavior (de Charms, 1968), *perceived competence* refers to the sense of being effective in one’s interactions and undertakings (White, 1959), and *perceived relatedness* refers to the sense of being connected to and cared for by important others (Baumeister & Leary, 1995). The three basic psychological needs form a core part of self-determination theory (Ryan & Deci, 2017), which posits that basic psychological need satisfaction is associated with a range of positive outcomes. Ample research in the academic domain supports this (e.g., Jang et al., 2016). In the social-emotional domain, need satisfaction is also critical (Ryan & Deci, 2017); however, only limited research has examined social-emotional variants of the basic psychological needs. Social-emotional need satisfaction as operationalized in the SEC School Model is now introduced.

*Perceived social-emotional autonomy* refers to the sense of having a say in how one thinks, acts, and feels in social and emotional situations and interactions (Collie, 2021; cf. de Charms, 1968). For perceived social-emotional competence, there are different types that apply to different capacities (e.g., for emotion regulation, for conflict resolution; Collie, 2022). Given the current’s study focus on prosocial motivation, the decision was made to focus on perceived social competence because it is also socially-oriented and thus conceptually aligned with prosocial motivation. *Perceived social competence* refers to students’ sense of feeling capable to interact with others including, for example, communicating clearly, listening considerately, cooperating effectively, and resolving disagreements constructively. Importantly, because perceived social competence involves self-perceptions relating to other-focused behaviors, it is particularly salient for supporting prosocial motivation—which also involves other-focused behaviors. Moreover, by focusing on social aspects (rather than both social and emotional aspects), the present study enabled a concentrated examination of the role of perceived social competence specifically in relation to prosocial motivation (and the outcomes).

Finally, for relatedness at school, which is inherently social and emotional in nature, it is important to consider both students and teachers. *Relatedness with students* refers to students’ perceptions that they care for and are cared for by other students (Baumeister & Leary, 1995). *Relatedness with teachers* is the same, but with respect to teachers.

A large body of research has highlighted the importance of need satisfaction with respect to academic outcomes (e.g., Jang et al., 2016). In addition, research has consistently highlighted the importance of relatedness for prosocial behavior (Wentzel et al., 2018) and well-being (Kim, 2021) among students. Although there is a growing body of work linking perceived emotional competence (for emotion regulation) with greater well-being among secondary school students (Metz et al., 2013) and university students (Bigman et al., 2016; Caprara et al., 2008), less research has considered perceived social competence. In one relevant study, Collie (2022) demonstrated that perceived social competence (for constructive conflict resolution) was associated with greater prosocial behavior. Finally, research examining perceived (academic) autonomy shows that it is associated with greater well-being among secondary school students (Garn et al., 2019). Ascertaining the extent to which a similar association occurs with perceived social-emotional autonomy remains an open empirical question.

Given this is a nascent area of research, important gaps in knowledge remain. In addition to the gaps described above, apparently no studies have considered all social-emotional need satisfaction factors within one study. This gap is important to address in order to determine the unique role that each basic psychological need plays as a mechanism of SEC. The present study, thus, examined the different types of social-emotional need satisfaction (autonomy, competence, and relatedness) as separate factors within the one model, alongside prosocial motivation and relevant outcomes.

### Linking social-emotional need satisfaction with prosocial motivation

Autonomous motivation in academic domains is well-established to be associated with positive outcomes, whereas the reverse is true for controlled motivation (Ryan & Deci, 2017). According to self-determination theory (Ryan & Deci, 2017), autonomous motivation involves highly self-determined behavior regulation (i.e., intrinsic, identified) that is characterized by volition and choice. In contrast, controlled motivation involves behavior regulation (i.e., introjected, external) that is characterized by pressures or demands that are perceived to be controlled by external means (Ryan & Deci, 2017). As noted above, the SEC School Model extrapolates this understanding of motivation to the social-emotional domains.

In the present study, prosocial motivation was examined. *Autonomous prosocial motivation* was operationalized as highly self-determined prosocial behavior regulation that involves: being motivated to undertake a prosocial behavior due to inherent interest or enjoyment (intrinsic regulation), or being motivated to undertake a prosocial behavior because the consequence is personally valued (identified regulation; Collie, 2021; Ryan & Deci, 2017). In contrast, *controlled prosocial motivation* was operationalized as prosocial behavior regulation that involves: being motivated to undertake a prosocial behavior to avoid guilt and shame (introjected regulation), or being motivated to undertake a prosocial behavior to avoid punishment (external regulation).

Countless studies looking at academic domains have demonstrated the positive role of need satisfaction for greater levels of autonomous motivation and lower levels of controlled motivation for schoolwork (Ryan & Deci, 2017). However, few studies have considered the extent to which these associations translate to the social-emotional domains and findings remain mixed. For example, Wentzel et al. (2007) demonstrated that perceived social competence was not uniquely associated with prosocial behavior among adolescents, whereas other studies among adults have provided support for these associations (e.g., Gagné, 2003; Weinstein & Ryan, 2010). Clearly, more research is needed. A key aim of the present study therefore was to examine social-emotional need satisfaction as a predictor of autonomous and controlled prosocial motivation. Social-emotional need satisfaction is essential for promoting internalization of key SEC-aligned values, norms, and beliefs (Collie, 2020; Ryan & Deci, 2017). In turn, this internalization is what leads to greater autonomous motivation and lower controlled motivation (Ryan & Deci, 2017).

Notably, the inclusion of controlled prosocial motivation in the present study moves beyond the conceptual model guiding the present study, the SEC School Model (Collie, 2020). Given its focus, the SEC School Model prioritizes the role of autonomous motivation for promoting socially and emotionally competent behaviors. However, Collie (2020; see also Collie, 2021) also acknowledges the importance of considering controlled motivation as it is likely to be associated with enactment of less adaptive behaviors. The present study thus tested the extent to which controlled prosocial motivation is negatively predicted by social-emotional need satisfaction. Importantly, recent research suggests the presence of a dual process in relation to autonomous and controlled motivation, whereby need satisfaction is more strongly associated with autonomous motivation than controlled motivation (e.g., Jang et al., 2016; cf. Donald et al., 2021). Based on this prior research (e.g., Gagné, 2003; Jang et al., 2016), the following hypothesis was posited:

#### **Hypothesis 1**

Social-emotional need satisfaction (i.e., perceived autonomy, competence, relatedness) is strongly and positively associated with autonomous prosocial motivation, and less strongly and negatively associated with controlled prosocial motivation.

### Supporting adaptive behavioral and well-being outcomes

The mechanisms of SEC are implicated in the behavioral and well-being outcomes experienced by students (Collie, 2020). In the present study, two behavioral outcomes were examined. *Prosocial behavior* reflects interpersonal actions that are undertaken to benefit others (Schroeder & Graziano, 2015), whereas *conduct problems* reflect antisocial behaviors such as disobeying rules, acting aggressively, and stealing (Bevilacqua et al., 2018). Two emotional well-being outcomes were also examined. *Positive affect* refers to experiences of positive emotions (e.g., inspired, active, attentive), whereas *negative affect* refers to experiences of negative emotions (e.g., afraid, nervous, upset; Diener & Emmons, 1984).

Empirical research yields emerging support for the role of autonomous prosocial motivation in relation to relevant behavioral outcomes. For example, secondary school students who report higher levels of autonomous prosocial motivation engage in fewer disruptive behaviors (Aelterman et al., 2019) and engage in fewer bullying behaviors (Roth et al., 2011). Researchers have also demonstrated the positive association between prosocial motivation and emotional well-being among adults (Weinstein & Ryan, 2010). There are mixed findings among students, however. For example, one study among adolescents found no association between prosocial motivation and prosocial behavior (Wentzel et al., 2007). Moving forward, more research is needed to extend the evidence base. In addition, it is important to build much-needed knowledge about controlled motivation and to extend knowledge of autonomous prosocial motivation in relation to other outcomes (i.e., conduct problems, emotional well-being) among school students. The present study, therefore, examined motivation in relation to the behavioral and well-being outcomes.

In addition, the current study also involved examination of direct associations and indirect associations (via motivation) from the need satisfaction factors to the outcomes. Prior research provides some support for direct associations between social-emotional need satisfaction and the outcomes. For example, a broader need satisfaction factor (not social-emotional) has been linked with greater positive affect among adolescents, but was unassociated with negative affect (Rodríguez-Meirinhos et al., 2020). Among children, perceived relatedness with teachers has been associated with greater prosocial behavior (Longobardi et al., 2020). Among adolescents, perceived social competence (for conflict regulation) has been linked with greater prosocial behavior (Collie, 2022; however, see Wentzel et al., 2007) and lower conduct problems in one study (Collie, 2022). Relatedly, perceived emotional competence (for emotion regulation) has been associated with greater prosocial behavior and emotional well-being among university students (Bigman et al., 2016; Caprara et al., 2008), and with fewer conduct problems among secondary school students (Parise et al., 2019). Importantly, these prior studies typically did not examine motivation, and so the present study provided the opportunity to test whether those direct associations remain when motivation is simultaneously examined. Turning to indirect associations, prior research in academic domains has shown evidence of these from need satisfaction to behavioral and well-being outcomes via academic motivation (Standage et al., 2005). The present study involved a test of whether indirect associations are also evident from social-emotional need satisfaction to the outcomes via prosocial motivation.

Taken together, a hypothesis was formed based on prior research (e.g., Aelterman et al., 2019) and the dual process where within-process associations (e.g., adaptive with adaptive) are stronger than cross-process associations (e.g., adaptive with maladaptive). More specifically, the associations between autonomous motivation and adaptive outcomes were anticipated to be positive, and those between controlled motivation and adaptive outcomes were anticipated to be negative and weaker in strength. The reverse was expected between autonomous motivation and maladaptive outcomes (weaker and negative) in comparison to those associations between controlled motivation and maladaptive outcomes (stronger and positive).

#### **Hypothesis 2a**

Autonomous motivation is positively associated with prosocial behavior and positive affect, and less strongly and negatively associated with conduct problems and negative affect. The reverse associations are anticipated for controlled motivation.

Given prior research showing that need satisfaction has direct associations with outcomes (e.g., Bigman et al., 2016), a related hypothesis was identified:

#### **Hypothesis 2b**

Social-emotional need satisfaction is positively associated with prosocial behavior and positive affect, and negatively associated with conduct problems and negative affect.

Finally, and based on prior research (Standage et al., 2005), it was anticipated that indirect associations would also be evident in the present study:

#### **Hypothesis 3**

Social-emotional need satisfaction is associated with the outcomes via autonomous motivation. Indirect associations via controlled motivation will be non-significant given the dual process hypothesis proposes weaker associations between need satisfaction and controlled motivation (see above).

### Background characteristics

Five background characteristics were included as covariate controls in the current study: gender, age, language background, attention-deficit/hyperactivity disorder (ADHD), and socio-economic status. These characteristics have been previously linked with the substantive factors examined in the present study and so served as controls to parse out the variance attributable to them. For example, female students typically exhibit more prosocial behaviors (Collie et al., 2019) and students with ADHD (the most prevalent neurodevelopmental disorder among children/adolescents in Australia; Lawrence et al., 2015) can struggle with emotion regulation (Bunford et al., 2020). Together, the background characteristics were included to control for variance attributable to them in the study.

## Study overview

The aim of the present study was to examine the extent to which social-emotional need satisfaction is associated with prosocial motivation, and whether both factors are (directly and/or indirectly) associated with behavioral and emotional well-being outcomes (while controlling for shared variance and background characteristics). Using structural equation modeling, students’ need satisfaction (i.e., perceived social-emotional autonomy, perceived social competence, perceived relatedness with students/teachers) was examined as a predictor of their prosocial motivation. Both the need satisfaction and motivation factors were then examined as predictors of parent/carer-reported behavioral outcomes (prosocial behavior, conduct problems) and student-reported emotional well-being (positive and negative affect). Finally, indirect associations among substantive factors were also tested. Figure 1 shows the hypothesized model.

## Method

### Sample and procedure

The sample comprised 408 secondary students from the states of Victoria (53%) and Queensland (47%) in Australia. Of the sample, 44% were female (the remainder were male) and the average age was 14 (*SD* = 1; range 13–16) years. Students were in grades 7 (14%), 8 (26%), 9 (22%), 10 (24%), 11 (12%), or 12 (2%) and most students (90%) spoke English at home. Ten percent (10%) had received a diagnosis of ADHD, which is slightly greater, but comparable to national reports (e.g., 7.4% of Australian children and adolescents; Lawrence et al., 2015). Students attended government (70%), Catholic (15%), or independent schools (15%) that were co-educational (90%), single-sex girls’ (6%), or single-sex boys’ schools (4%). Socio-economic status (SES) was assessed with home postcode and the Australian Bureau of Statistics (ABS) index of relative socio-economic advantage and disadvantage (ABS, 2018). For the present study, the average SES was 1002 (SD = 67), which is around the national average (i.e., *M* = 1000, *SD* = 100; ABS, 2018). Parents/carers who completed part of the questionnaire were 69% female, 30% male, and < 1% non-binary.

Data were collected via an online questionnaire over two weeks in April–May, 2021 during the second quarter of the school year in Australia. This timeframe occurred during the COVID-19 pandemic. Importantly, there were no cases of community transmission in the states of Victoria or Queensland at the time (O’Brien, 2021), and students were attending school in-person as usual. Participant recruitment occurred through Qualtrics and their market research partners, which have contact details of a broad sample of the Australian population. Parents (or carers) of adolescents had previously indicated their interest in receiving information about studies run for parents and children. This online approach to recruitment enabled sampling from across two states in Australia, and allowed data collection from both parents and their adolescent student. The study invitation was sent to potential respondents via email or app notification. Parents opened the questionnaire URL to participate and then answered screening questions to check they had an adolescent aged 13 to 16 years old who was attending an Australian school in-person. Parents who did not pass the screening questions were exited from the questionnaire. If parents passed the screening questions, they were asked questions about their adolescent aged 13–16 years of age (if they had more than one adolescent within the age range, they were asked to choose one child). Following this, parents were then asked to pass their mobile device or computer to the same adolescent, who then completed the student section of the questionnaire. Both parents and adolescents were asked to provide consent at the start of their sections of the questionnaire. Respondents who completed the survey very quickly (less than 1/3 of the median time for completion of the questionnaire) or who answered the same way across many items in a row (80% of the survey) were removed from the final sample. IP addresses were cross-referenced with respondents’ socio-demographic characteristics to ensure there were no duplicate respondents. Of the respondents who passed the screening question, the response rate for the study was 74%. Approval from the Institutional Review Board was received for the study.

### Measures

Unless stated otherwise, items were scored from 1 (disagree strongly) to 7 (agree strongly). The behavioral outcomes were parent-reported. All other scales were self-reported by adolescents.

#### Social-emotional need satisfaction

New scales were developed to assess perceived autonomy and social competence. Scale development was based on theoretical understanding of basic psychological needs (Ryan & Deci, 2017; White, 1959) and SEC conceptualizing (Collie, 2020). *Perceived social-emotional autonomy* was assessed with four items: “I have choice and freedom in how I behave at school,” “My decisions for how I interact with others at school are what I really choose,” “My choices for how I act at school reflect who I really am,” and “I am free to choose the person I want to be at school.” *Perceived social competence* was assessed with four items: “At school, I can communicate my ideas clearly in group work,” “I can listen considerately to other students’ ideas in group work,” “I am confident I can cooperate well with most students in group work,” and “I feel capable at resolving disagreements at school by being respectful to the other people involved.” *Perceived relatedness with students* was assessed with Collie and Martin's (2021) adaptation of items from Chen et al.’s (2015) Basic Psychological Need Satisfaction and Frustration Scale. Adaptations involved changing “people” to “students” and adding “at my school” (e.g., “I feel that the students I care about at my school also care about me”). *Perceived relatedness with teachers* was assessed with four items developed here and derived from prior work (e.g., Martin & Marsh, 2008): “I like my teachers,” “My relationships with my teachers are positive,” “I get on well with my teachers,” and “I feel close and connected with my teachers.”).

For reliability, McDonald’s omega coefficients were calculated from a confirmatory factor analysis (CFA) involving all substantive variables and were adequate at ω = 0.79 for social-emotional autonomy, ω = 0.84 for social competence, ω = 0.86 for relatedness with students, and ω = 0.91 for relatedness with teachers. Additional evidence of validity is presented below, including measurement invariance tests.

#### Prosocial motivation

New scales based on conceptual guidance from self-determination theory (Ryan & Deci, 2017) were developed for assessing students’ prosocial motivation for helping others, being nice, and being caring (e.g., “I put effort into helping students who are hurt or upset at school…”). Students responded to the item stems by indicating their agreement with different types of behavior regulation (12 items): intrinsic regulation (e.g., “…because I enjoy being kind”), identified regulation (e.g., “…because I believe this is important to do”), introjected regulation (e.g., “…because otherwise I will feel like a bad person”), and external regulation (e.g., “…because I risk getting in trouble if I don’t”). In preliminary analyses, it was apparent that the introjected and external regulation were only moderately correlated (see Table 1) and so these factors were modeled separately in analyses. As anticipated based on other research on prosocial motivation (Longobardi et al., 2020), the intrinsic and identified regulation factors were highly correlated and so these items were loaded onto one factor representing autonomous motivation. Reliability was adequate for the three factors: ω = 0.91 for autonomous motivation, ω = 0.71 for introjected regulation, and ω = 0.75 for external regulation. Additional evidence of validity evidence is presented below.

**Table 1:** Reliability estimates, descriptive statistics, and factor loadings

	Omega (ω)	*M*	*SD*	Standardized Factor Loadings *M* (Range)
*Need satisfaction*				
Social-emotional autonomy	.79	5.17	0.98	.69 (.67–.73)
Social competence	.84	5.27	1.03	.76 (.72–.78)
Relatedness with students	.86	5.38	1.01	.78 (.68–.85)
Relatedness with teachers	.91	5.16	1.14	.84 (.82–.87)
*Prosocial motivation*				
Autonomous motivation	.91	5.74	0.92	.79 (.74–.84)
Introjected regulation	.71	4.47	1.25	.67 (.57–.78)
External regulation	.75	3.39	1.30	.70 (.62–.83)
*Outcomes*				
Prosocial behavior	.76	7.15	2.19	.62 (.57–.75)
Conduct problems	.75	1.96	2.04	.61 (.50–.71)
Positive affect	.76	3.50	0.65	.62 (.46–.73)
Negative affect	.82	2.41	0.67	.68 (.61–.71)

*Note.* The omega coefficients for prosocial behavior and conduct problems were calculated from two congeneric CFAs. In analyses, an error-adjusted sum score for these constructs was used

#### Behavioral outcomes

Parent (or carer) reports of students’ prosocial behavior and conduct problems were assessed using the Strengths and Difficulties Questionnaire (Goodman, 1997). Parents responded to items based on their adolescent’s behavior over the past six months. For both factors, items were scored 0 (not true), 1 (somewhat true), or 2 (certainly true). Five items assessed prosocial behavior (e.g., “considerate of other people’s feelings”) and another five items assessed conduct problems (e.g., “often fights with other youth or bullies them”). Reliability was adequate for both scales: ω = 0.76 for prosocial behavior and at ω = 0.75 for conduct problems. The omega coefficients were calculated from separate congeneric CFAs involving items in each scale. In analyses, an (error-adjusted) sum score was used for each behavioral outcome (Goodman, 1997). Prior research has demonstrated the appropriateness of using parent-reports from the SDQ among Australian adolescents (Mellor, 2005). Moreover, the descriptive statistics (*M, SD*) in the present study are very similar to those obtained in prior Australian research using the same measure (Collie, 2022; Mellor, 2005).

#### Well-being outcomes

Positive and negative affect were assessed using the International Positive and Negative Affect Schedule Short Form (Thompson, 2007). Students were asked to consider how they generally feel in relation to five positive emotions (i.e., determined, attentive, alert, inspired, and active) and five negative emotions (i.e., afraid, nervous, upset, ashamed, and hostile). Items were scored on a scale from 1 (Never) to 5 (Always). Reliability was adequate for positive affect (ω = 0.76) and negative affect (ω = 0.82). Moreover, the descriptive statistics in the current study are very similar to those obtained in other research among adolescent populations using the same scale (e.g., Collie, 2022; Quinlan et al., 2015).

#### Covariates

Gender was coded 0 for male students and 1 for female students. Age was measured in years. Language background was coded 0 for English spoken at home and 1 for a non-English speaking background. Parents reported on ADHD diagnosis: 0 for no ADHD diagnosis; 1 for an ADHD diagnosis. Socio-economic status (SES) was measured by comparing home postcode (which parents reported) with the ABS (2018) index of relative socio-economic advantage and disadvantage, where a higher score means a higher SES.

### Data Analysis

Mplus 8.6 (Muthén & Muthén, 2021) was used for all analyses. Mean and standard deviations were calculated in preliminary analyses. Main analyses were conducted with confirmatory factor analysis (CFA) and structural equation modeling (SEM) using the robust maximum likelihood (MLR) estimator and full information maximum likelihood to handle missing data (≤ 1%). CFA and SEM model fit was assessed with the comparative fit index (CFI), Tucker Lewis index (TLI), and the root-mean-square error of approximation (RMSEA). CFI and TLI values of ≥ 0.90 indicate adequate fit (Hu & Bentler, 1999). RMSEA values of ≤ 0.08 indicate adequate fit (Hu & Bentler, 1999).

A CFA involving all covariates and substantive factors provided an assessment of the factor structure of latent variables, enabled calculation of reliability estimates using McDonald’s omega coefficient, and provided latent correlations among factors. Because several of the scales in the present study were newly developed, measurement invariance tests (with multigroup CFA) were used to assess whether the items functioned similarly across major student subgroups (i.e., by gender, age, and SES; see Supplementary Materials for details).

In the CFAs and SEM, latent factors were specified for substantive constructs. The exception to this involved the behavioral outcomes, which are typically modeled as sum scores (Goodman, 1997). In the present study, error-adjusted sum scores were estimated to provide some control for measurement error. These two variables were specified in modeling with the loading constrained to 1 and the residual constrained using the following equation: σ^2^ * (1- ω), where σ^2^ is the variance and ω is the reliability of a variable (Brown, 2006). Estimates of variance and reliability (omega coefficient) for the behavioral outcomes were taken from separate congeneric CFAs. Covariates were estimated with loading set to 1 and residual set to 0 in all models.

SEM examined the structural paths as per the hypothesized model (see Fig. 1): need satisfaction was entered as a predictor of motivation. In turn, the need satisfaction and motivation factors were entered as predictors of the four outcomes. Covariates served as controls for all constructs. Constructs at the same point in the model were freely allowed to covary to control for shared variance. After testing the main (direct) paths, indirect associations among factors were examined. A non-parametric bootstrapping approach was used for this (1,000 draws; Shrout & Bolger, 2002).

## Results

Table 1 displays the mean, standard deviation, and factor loading mean and range for each substantive factor in the hypothesized model. Measurement invariance tests supported the equivalence of loadings and intercepts across major student subgroups (by gender, age, and SES; see Supplementary Materials for details). The CFA yielded adequate fit: χ^2^(832) = 1248.82, *p* < 0.001, RMSEA = 0.035, CFI = 0.94, TLI = 0.93. Correlations from the CFA are shown in Table 2 and generally followed the dual process model (within-process factors positively correlated; between-process factors negatively correlated).

**Table 2:** Latent correlations from confirmatory factor analysis

	1	2	3	4	5	6	7	8	9	10	11	12	13	14	15
*Covariates*															
1. Gender															
2. Age	− .04														
3. Lang. background	.01	.01													
4. ADHD	− .06	.01	− .09**												
5. SES	− .11*	.05	.13**	− .09*											
*Need satisfaction*															
7. Social-emotional autonomy	.06	− .02	.12*	− .10	.01										
Social competence	.08	.06	.13*	− .24**	.06	.70**									
8. Rel. with students	.07	.01	.09	− .19**	.03	.68**	.76**								
9. Rel. with teachers	.08	− .02	.12*	− .17**	.06	.74**	.76**	.72**							
*Prosocial motivation*															
10. Autonomous	.09	− .10	.05	− .11	.04	.58**	.65**	.56**	.65**						
11. Introjected	− .03	− .08	.10	.03	.02	.15*	.19*	.20*	.23**	.37**					
12. External	− .07	.08	− .04	.12*	− .10	− .18**	− .26**	− .13*	− .23**	− .38**	.37**				
*Outcomes*															
13. Prosocial behavior	.14**	− .01	.01	− .12*	− .02	.41**	.50**	.45**	.46**	.62**	.21**	− .32**			
14. Conduct problems	− .07	− .05	− .09**	.40**	− .06	− .42**	− .52**	− .43**	− .48**	− .38**	− .06	.25**	− .44**		
15. Positive affect	.02	.09	.14*	− .19**	.07	.59**	.68**	.61**	.60**	.50**	.06	− .27**	.48**	− .52**	
16. Negative affect	.21**	− .09	− .04	.30**	− .03	− .50**	− .49**	− .36**	− .42**	− .24**	.16*	.22**	− .16**	.54**	− .52**

*Note.* Gender was coded 0 for male students and 1 for female students. Language background was coded as 0 for English only and 1 for a non-English speaking background. ADHD was coded as 0 for no ADHD diagnosis and 1 for an ADHD diagnosis. Lang. background = Language background. ADHD = Attention-deficit/hyperactivity disorder. SES = socio-economic status. Rel. = Relatedness

^*^ *p* ≤ .05; ** *p* ≤ .01

**Fig. 2:**
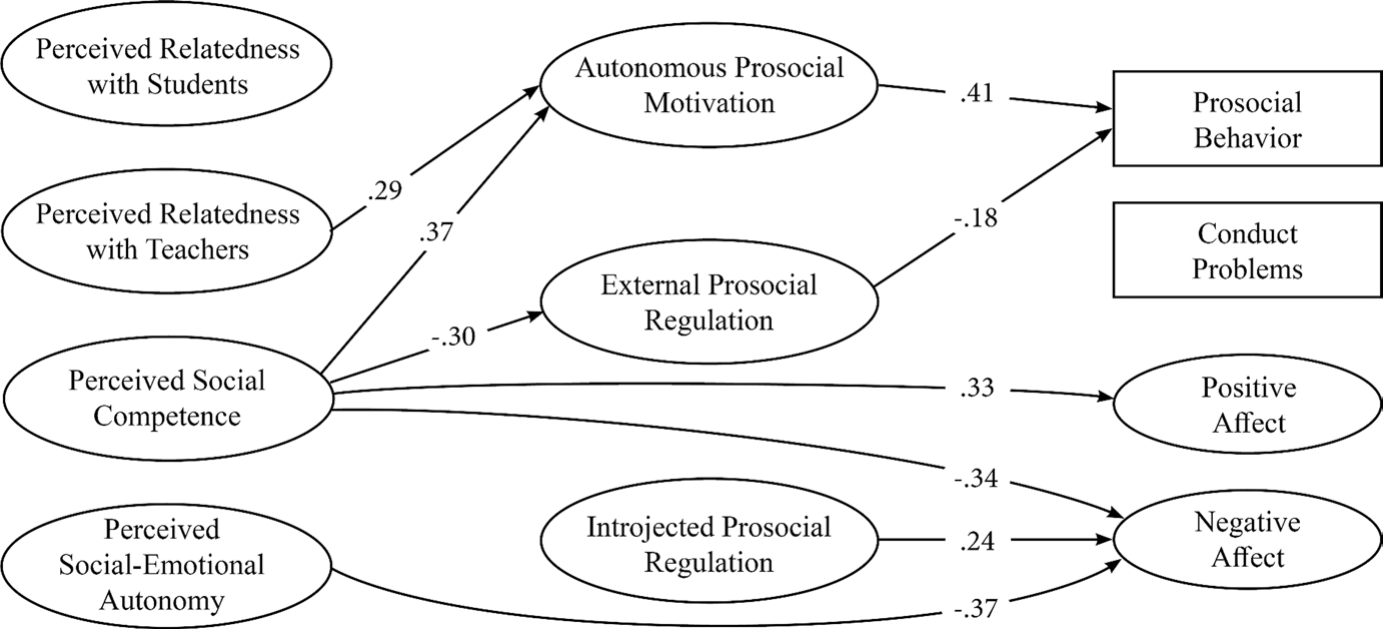
Standardized beta estimates from structural equation* model. Note*. All paths significant at *p* < .05. Covariates not shown for clarity; however, all results are shown in Table 3

The SEM yielded adequate fit: χ^2^(832) = 1248.82, *p* < 0.001, RMSEA = 0.035, CFI = 0.94, TLI = 0.93. Table 3 shows standardized beta estimates, *R*^*2*^, and adjusted *R*^*2*^. Figure 2 displays significant findings among substantive factors (for results involving covariates, see Supplementary Materials). Perceived social-emotional autonomy was associated with lower negative affect. Perceived social competence was associated with greater autonomous motivation, lower external regulation, greater positive affect, and lower negative affect. Perceived relatedness with teachers was associated with greater autonomous motivation. In turn, autonomous motivation was associated with greater prosocial behavior, whereas external regulation was associated with lower prosocial behavior. Introjected regulation was associated with greater negative affect. The model explained substantial variance in autonomous motivation and all outcomes (*R*^2^ and adjusted *R*^2^ > 35%), but much less in the two forms of controlled motivation (~ 10%). There was one significant indirect association: perceived social competence → autonomous motivation → prosocial behavior (β = 0.15, *SE* = 0.07, *p* = 0.023, 95% CI [0.02, 0.28]).

**Table 3:** Standardized beta estimates from the structural equation model

	Need satisfaction				Prosocial Motivation			Outcomes			
	Social-emotional autonomy	Social competence	Rel. with students	Rel. with teachers	Autonomous	Introjected	External	Prosocial behavior	Conduct problems	Positive affect	Negative affect
*Covariates*											
Gender	.05	.08	.06	.08	.03	− .05	− .05	.08*	− .01	− .04	.28**
Age	− .02	.07	.01	− .02	− .11**	− .09	.10	.05	− .05	.08	− .04
Lang. background	.12*	.10	.07	.10	− .04	.09	.01	− .05	− .01	.05	.03
ADHD	− .08	− .22**	− .18**	− .15*	.04	.08	.05	− .02	.30**	− .04	.21**
SES	− .01	.04	.01	.05	.01	− .01	− .08	− .05	.01	.02	.03
*Need satisfaction*											
Social-emotional autonomy					.10	− .10	− .02	− .01	− .08	.14	− .37**
Social competence					.37**	.04	− .30*	.08	− .22	.33**	− .34**
Rel. with students					.01	.10	.22	.14	− .02	.17	.13
Rel. with teachers					.29*	.21	− .12	− .03	− .16	.08	− .08
*Prosocial motivation*											
Autonomous								.41**	− .01	.06	.08
Introjected								.10	− .02	− .07	.24*
External								− .18*	.11	− .07	.01
*R-squared*	3%	8%	4%	5%	50%	8%	11%	44%	39%	52%	48%
*Adjusted R-squared*	2%	7%	3%	4%	49%	7%	10%	43%	38%	51%	47%

Gender was coded 0 for male students and 1 for female students. Language background was coded as 0 for English only and 1 for a non-English speaking background. Lang. background = Language background. ADHD was coded as 0 for no ADHD diagnosis and 1 for an ADHD diagnosis. ADHD = Attention-deficit/hyperactivity disorder. SES = socio-economic status

* *p* ≤ .05; ** *p* ≤ .01

## Discussion

This study examined students’ social-emotional need satisfaction, prosocial motivation, and their behavioral and well-being outcomes. Findings revealed that perceived social-emotional autonomy was associated with lower negative affect. Perceived social competence was associated with greater autonomous motivation and positive affect, and lower external regulation and negative affect. Relatedness with teachers was also associated with greater autonomous prosocial motivation. In turn, autonomous motivation was associated with greater prosocial behavior. Introjected and external regulation were associated in different ways with the outcomes. Introjected regulation predicted greater negative affect, whereas external regulation predicted lower prosocial behavior. Key findings are discussed below.

### The role of social-emotional need satisfaction

This is the first study to examine the two types of mechanisms simultaneously—social-emotional need satisfaction and social-emotional motivation—and thus provides important empirical support for theoretical work in the area. In general, the findings demonstrate that social-emotional need satisfaction lays a foundation for prosocial motivation (Hypothesis 1) and the outcomes (Hypothesis 2). However, there were some interesting nuances.

Looking first at perceived social competence, this was a highly salient factor in the SEM given its unique associations with numerous other factors: greater prosocial motivation, lower external prosocial regulation, greater positive affect, and lower negative affect. These findings align with foundational understanding from self-determination theory (Ryan & Deci, 2017) and conceptualizing in the area of SEC (Collie, 2020). Importantly, these findings provide empirical support for and extend prior work by revealing the centrality of perceived social competence. More precisely, the association with autonomous prosocial motivation was as hypothesized (Hypothesis 1) and likely occurred because individuals who feel more capable in relation to their social interactions come to internally endorse the values and beliefs associated with prosocial interactions—and thus are more autonomously motivated for prosocial behavior as a result (Collie, 2020; Ryan & Deci, 2017).

High levels of perceived social competence also mean that students are less likely to feel externally pressured in their prosocial motivation—explaining the negative association with external prosocial regulation. Notably, the negative association between perceived social competence and external regulation was moderate in strength, rather than weak as anticipated (Hypothesis 1). Thus, perceived social competence appears to play a unique and sizeable role both for autonomous prosocial motivation and external prosocial regulation. Given this is the first study to examine these associations, it will be important to see if they replicate among other samples and studies.

In terms of the well-being outcomes, perceived competence is known to underlie well-being (Ryan & Deci, 2017). Perceived competence leads to positive affect because it means individuals feel self-confident and optimistic in relation to their interactions (White, 1959). As anticipated (Hypothesis 2b), the present finding suggests a similar process occurs in the social domains. Indeed, perceived social competence likely helps students to avoid the self-doubt that can come from low perceived competence around social interactions (e.g., anxiety; Bornstein et al., 2010).

In line with Hypothesis 1, there was a positive association between relatedness with teachers and autonomous prosocial motivation. Ample prior research has demonstrated that relatedness with teachers is crucial for academic outcomes among students (e.g., Martin & Collie, 2019). The present study shows that relatedness with teachers is also important for autonomous prosocial motivation. When students feel a positive bond with their teachers, this likely helps them to internalize the values associated with caring and respectful relationships—and, in turn, this translates to autonomous prosocial motivation (Collie, 2020; Ryan & Deci, 2017). This finding is important in light of other research that has suggested teacher-student relationship quality can decrease in adolescence (Hughes & Cao, 2018). Thus, the present study provides additional evidence of the importance of positive teacher-student relationships among adolescent students.

An unexpected finding was that relatedness with students was not associated with any prosocial motivation factors (Hypothesis 1). This contrasts prior research showing that relatedness with students was positively associated with prosocial behavior as reported by peers (but not as reported by teachers; Wentzel et al., 2018). In the present study, the unexpected finding was the case in the SEM, where controls for shared variance were in place, but not in the CFA. In the CFA, bivariate correlations demonstrated that relatedness with students was positively associated with autonomous prosocial motivation and prosocial behavior (among other factors). Perhaps these findings did not carry over to the SEM because perceived social competence accounted for the aspects of peer interaction that are relevant to prosocial motivation, leaving aspects not uniquely relevant to prosocial motivation (e.g., having fun with friends). Given Wentzel et al. (2018) did not measure perceived social competence in their study, more research is needed to examine if this finding holds in other samples and whether relatedness with students is salient in relation to other types of social-emotional motivation (e.g., for emotion regulation).

Partial support for Hypothesis 2b was found in relation to perceived social-emotional autonomy, which was associated with lower negative affect, but not associated with positive affect (or the behavioral outcomes). Perceived autonomy has been specifically emphasized as being key to avoiding mental health issues (Ryan & Deci, 2017). It may be that feeling free to choose how one acts, thinks, and feels at school means that students experience fewer negative emotions because they feel less pressured or controlled to be someone different (Ryan & Deci, 2017). Of note, these findings contradict Rodríguez-Meirinhos et al.’s (2020) study, which demonstrated that a broader factor of need satisfaction was associated with positive affect, but not negative affect. Perhaps this difference occurred because the present study focused on social-emotional autonomy (rather than general autonomy) and on the specific types of need satisfaction (not a broader factor). Future research that endeavors to disentangle this finding and why it occurred is needed.

Taken together, these findings provide evidence of the importance of need satisfaction in the social-emotional domains—and, in particular, for prosocial motivation, prosocial behavior, and emotional well-being. Of note, the SEM explained greater variance in autonomous prosocial motivation than in the two types of controlled prosocial motivation, which likely relates to the dual process hypothesis. More precisely, given the predictors were adaptive in nature, it is understandable they explained more variance in the adaptive outcomes than maladaptive outcomes. Going forward, research examining social-emotional need frustration (i.e., perceived pressure, incompetence, loneliness) is important to test if those factors play a stronger role in predicting controlled prosocial motivation.

### Links with the outcomes

Autonomous prosocial motivation was positively associated with (parent-reported) prosocial behavior as anticipated (Hypothesis 2a). This finding differs from an early study in the area (Wentzel et al., 2007), but aligns with more recent research (e.g., Roth et al., 2011). According to conceptualizing (Collie, 2020; Ryan & Deci, 2017), when students are motivated to be prosocial due to inherent satisfaction or because they value the consequences of such behavior, this means they are more likely to engage in prosocial behavior.

As hypothesized, the reverse association was found for external prosocial regulation (Hypothesis 2a). Students who were motivated to be prosocial due to fear of punishment were less likely to exhibit prosocial behavior—possibly because external prosocial regulation involves no internal endorsement of the consequences of prosocial behaviors (Collie, 2020; Ryan & Deci, 2017). This second finding differs from earlier work (Wentzel et al., 2007)—again, highlighting the need for more research to understand the contexts in which this finding does or does not occur. Notably, the finding suggests there may be merit in efforts to help reduce external prosocial motivation among students, alongside efforts to boost autonomous prosocial motivation. In addition, the finding provides support for the dual process hypothesis. Namely, the absolute value of the association between autonomous motivation and prosocial behavior was stronger than that between external regulation and prosocial behavior (Hypothesis 2a).

Contrary to expectations, none of the need satisfaction or motivation factors were associated with conduct problems (Hypotheses 2a and 2b). It is likely this occurred due to the controls for shared variance in the SEM—indeed, there were significant bivariate correlations between conduct problems and the other factors in the CFA. Perhaps in the SEM after parsing out the negative affect that can often go hand-in-hand with conduct problems (Memmott-Elison et al., 2020), being motivated to help others (e.g., for enjoyment or to avoid guilt or getting in trouble) is no longer relevant to conduct problems. Future research that examines other types of social-emotional motivation is important to disentangle this finding further.

A related finding was that there were no direct associations between the need satisfaction factors and prosocial behavior, which was counter to expectations (Hypothesis 2b)—all associations occurred via the motivation factors. This finding extends prior work that has examined need satisfaction and prosocial behavior without motivation (e.g., Collie, 2022), and highlights the importance of considering the role of motivation. More precisely, it appears that perceived social competence on its own is not necessarily enough for fostering the enactment prosocial behavior. Instead, it appears that perceived social competence needs to be accompanied by autonomous prosocial motivation.

As anticipated (Hypothesis 2a), introjected prosocial regulation was associated with greater negative affect. Being motivated to be prosocial because one wants to avoid negative self-focused emotions (e.g., guilt, shame) may actually lead individuals to experience more negative emotions. This is because introjected regulation involves feeling pressured or controlled (albeit by oneself) and is tied up with the perception that one’s self-worth is conditional (Ryan & Deci, 2017).

Finally, the SEM explained substantial amounts of variance in the outcomes. The values were slightly higher for the adaptive outcomes than the maladaptive outcomes—possibly relating to the dual process hypothesis once again. The variance explained provides important preliminary evidence of the value of addressing the need satisfaction and motivation factors in intervention efforts (discussed below).

### Indirect associations

There was one significant indirect association showing that perceived social competence was associated with prosocial behavior via autonomous motivation (Hypothesis 3). As noted earlier, research looking at academic factors has shown evidence of indirect associations from need satisfaction to outcomes via academic motivation (Standage et al., 2005). The present study provides evidence to suggest this carries over to the social-emotional domains. Moreover, this finding further underscores the salient role of prosocial motivation in linking need satisfaction and prosocial behavior. Further still, the finding suggests that perceived social competence may be an avenue to foster prosocial behavior.

### Implications for practice

Social and emotional learning programs are one way that schools can support students’ mechanisms and manifestations of SEC. Social and emotional learning programs involve curriculum designed to teach skills and values associated with SEC, and efforts to promote a supportive school climate (Weissberg et al., 2015). Research shows that effective social and emotional learning programs can boost students’ prosocial behavior and emotional well-being (Jagers et al., 2015). In addition, emerging research is showing that social and emotional interventions are also linked with the mechanisms of SEC. For example, a mindfulness program was found to boost adolescents’ self-efficacy for emotion regulation (Metz et al., 2013), and a social and emotional intervention that affirmed students’ autonomy had a stronger impact on reducing conduct problems than the same program without the autonomy affirmation (de Mooij et al., 2022).

Need-supportive teaching has been identified as another avenue that is linked with need satisfaction, motivation, and positive outcomes (Cheon et al., 2018; Collie, 2022). Need-supportive teaching comprises autonomy-support, competence-support, and relatedness-support. Autonomy-supportive teaching practices entail providing rationales for why it is important to be a caring member of the school community, paying attention to and acknowledging students’ viewpoints on how they are feeling, providing choices where possible for how students can manage their social-emotional experiences, and encouraging students’ involvement in relation to the creation or refinement of classroom or school rules and norms (Cheon et al., 2018; Collie, 2022; Roth et al., 2011). Competence-supportive teaching practices include providing clear goals, norms, and expectations for social-emotional interactions, and offering constructive feedback for how students can be considerate in responding to others during group discussions (Collie, 2020, 2022). Relatedness-supportive practices include the suggestions above, but also showing interest in students and their development, dedicating resources and time to students, and being attuned to what students need in their learning (Collie, 2020; Skinner & Belmont, 1993). Together, these are some practices that teachers may want to embed in their teaching to support students’ SEC.

### Limitations and future directions

The current study should be interpreted within the constraints of several limitations. First, most variables were assessed using student self-reports. Although this is an appropriate means for assessing intrapsychic constructs, it will be important to conduct research with multiple waves of data to address concerns about common-source bias. Importantly, however, a strength of the present study was that the behavioral outcomes were reported by parents (or carers). In future, it will be important to triangulate this with reports from teachers about students’ behavior within the classroom. Second, the data were cross-sectional in nature, which means that causal ordering is not possible to test. The hypothesized model (and construct ordering within it) was firmly derived from theory. Nonetheless, additional research with longitudinal and experimental designs is needed to test the direction of associations. Third, it is not possible to rule out sampling bias given the participants had signed up to receive information about research studies involving adolescents. Research using other samples and with other recruitment methods are needed to replicate the present study’s findings. At the same time, the means and standard deviations for several factors were very similar to other studies using the same scales, providing support for the representativeness of the sample. Fourth, the study included several background characteristics. Because this is one of the first studies examining this collection of variables, these background characteristics were examined only as covariate controls. Going forward, more in-depth examinations of these covariates will be important to ascertain whether there are any differences in associations by subpopulations. Fifth, perceived social competence formed the focus in the present study to precisely understand its role in prosocial motivation (and the outcomes). Going forward it will be important to test the role of perceived emotional competence alongside perceived social competence in relation to prosocial and other types of social-emotional motivation. Sixth, ADHD status was examined as a covariate given that it is the most prevalent neurodevelopmental disorder among school students. It will be important to consider other neurodevelopmental disorders that are less prevalent, but still relevant, in future research.

## Conclusion

The aim of the present study was to extend knowledge of SEC by examining two key mechanisms, along with important behavioral and well-being outcomes. The findings revealed the role of social-emotional need satisfaction and prosocial motivation as two central mechanisms in SEC. Namely, the need satisfaction factors played unique roles in relation to prosocial motivation and, in turn, prosocial behavior and emotional well-being. Taken together, the findings provide evidence for the importance of considering both social-emotional need satisfaction and social-emotional motivation in research and when developing interventions designed to promote adaptive social-emotional behaviors among students.

## Supplementary Information

Below is the link to the electronic supplementary material.

Supplementary file1 (DOCX 20 kb)

## Declarations:

### Data

The Institutional Research Board ethics approval received for these data does not allow for data sharing.

## References

[CR1] Aelterman N, Vansteenkiste M, Haerens L (2019). Correlates of students’ internalization and defiance of classroom rules: A self-determination theory perspective. British Journal of Educational Psychology.

[CR2] Australian Bureau of Statistics (ABS) (2018). *Technical paper: Socio-economic indexes for areas (SEIFA) 2016.* ABS. https://www.abs.gov.au/

[CR3] Bandura A (1997). Self-efficacy: The exercise of control.

[CR4] Baumeister RF, Leary MR (1995). The need to belong: Desire for interpersonal attachments as a fundamental human motivation. Psychological Bulletin.

[CR5] Bevilacqua L, Hale D, Barker ED, Viner R (2018). Conduct problems trajectories and psychosocial outcomes: A systematic review and meta-analysis. European Child and Adolescent Psychiatry.

[CR6] Bigman YE, Mauss IB, Gross JJ, Tamir M (2016). Yes I can: Expected success promotes actual success in emotion regulation. Cognition and Emotion.

[CR7] Bornstein MH, Hahn C, Haynes OM (2010). Social competence, externalizing, and internalizing behavioral adjustment from early childhood through early adolescence: Developmental cascades. Development and Psychopathology.

[CR8] Brown TA (2006). Confirmatory factor analysis for applied research.

[CR9] Bunford N, Dawson AE, Evans SW, Ray AR, Langberg JM, Owens JS, DuPaul GJ, Allan DM (2020). The difficulties in emotion regulation scale-parent report: A psychometric investigation examining adolescents with and without ADHD. Assessment.

[CR10] Caprara GV, Di Giunta L, Eisenberg N, Gerbino M, Pastorelli C, Tramontano C (2008). Assessing regulatory emotional self-efficacy in three countries. Psychological Assessment.

[CR11] de Charms RC (1968). Personal causation: The internal affective determinants of behavior.

[CR12] Chen B, Vansteenkiste M, Beyers W, Boone L, Deci E, Van DK, Duriez B, Lens W, Matos L, Mouratidis A, Ryan R, Sheldon K, Soenens B, VanPetegem S, Verstuyf J (2015). Basic psychological need satisfaction, need frustration, and need strength across four cultures. Motivation and Emotion.

[CR13] Cheon SH, Reeve J, Ntoumanis N (2018). A needs-supportive intervention to help PE teachers enhance students' prosocial behavior and diminish antisocial behavior. Psychology of Sport and Exercise.

[CR14] Collie RJ (2020). The development of social and emotional competence at school: An integrated model. International Journal of Behavioral Development.

[CR15] Collie RJ (2022). Instructional support, perceived social and emotional competence, and students’ behavioral and emotional well-being outcomes. Educational Psychology.

[CR17] Collie, R.J. (2021). Motivation theory and its yields for promoting students’ social and emotional competence. In N. Yoder and A. Skoog-Hoffman (Eds.), *Motivating the SEL field forward through equity* (Advances in motivation and achievement, Vol 21, pp. 41–58). Emerald Publishing. 10.1108/S0749-742320210000021004

[CR18] Collie, R.J., & Martin, A.J. (2021). *Adaptations to the Chen et al.’s (2015) Basic Psychological Need Satisfaction and Frustration Scale*. Educational Psychology Research Group, University of New South Wales.

[CR16] Collie RJ, Martin AJ, Nassar N, Roberts CL (2019). Social and emotional behavioral profiles in kindergarten: A population-based latent profile analysis and links to socio-educational characteristics and later achievement. Journal of Educational Psychology.

[CR41] de Mooij B, Fekkes M, van den Akker AL, Vliek L, Scholte RHJ, Overbeek G (2022). Does affirming children’s autonomy and prosocial intentions help? A microtrial into intervention component effects to improve psychosocial behavior. Journal of School Psychology.

[CR19] Diener E, Emmons RA (1984). The independence of positive and negative affect. Journal of Personality and Social Psychology.

[CR20] Domitrovich CE, Durlak JA, Staley KC, Weissberg RP (2017). Social-emotional competence: An essential factor for promoting positive adjustment and reducing risk in school children. Child Development.

[CR21] Donald JN, Bradshaw EL, Conigrave JH, Parker PD, Byatt LL, Noetel M, Ryan RM (2021). Paths to the light and dark sides of human nature: A meta-analytic review of the prosocial benefits of autonomy and the antisocial costs of control. Psychological Bulletin.

[CR22] Eccles JS, Midgley C, Wigfield A, Buchanan CM, Reuman D, Flanagan C, Mac Iver D (1993). Development during adolescents: The impact of stage-environment fit on young adolescents’ experiences in schools and in families. American Psychologist.

[CR23] Gagné M (2003). The role of autonomy support and autonomy orientation in prosocial behavior engagement. Motivation and Emotion.

[CR24] Garn AC, Morin AJS, Lonsdale C (2019). Basic psychological need satisfaction toward learning: A longitudinal test of mediation using bifactor exploratory structural equation modeling. Journal of Educational Psychology.

[CR25] Goodman R (1997). The strengths and difficulties questionnaire: A research note. Journal of Child Psychology and Psychiatry.

[CR26] Hu LT, Bentler PM (1999). Cutoff criteria for fit indexes in covariance structure analysis: Conventional criteria versus new alternatives. Structural Equation Modelling: A Multidisciplinary Journal.

[CR27] Hughes JN, Cao Q (2018). Trajectories of teacher-student warmth and conflict at the transition to middle school: Effects on academic engagement and achievement. Journal of School Psychology.

[CR28] Jagers R, Harris A, Skoog A, Durlak JA, Weissberg RP, Gullotta TP (2015). A review of classroom-based SEL programs at the middle school level. Handbook of social and emotional learning: Research and practice.

[CR29] Jang H, Kim EJ, Reeve J (2016). Why students become more engaged or more disengaged during the semester: A self-determination theory dual-process model. Learning and Instruction.

[CR31] Kim J (2021). The quality of social relationships in schools and adult health: Differential effects of student-student versus student-teacher relationships. School Psychology.

[CR33] Lawrence, D., Johnson, S., Hafekost, J., Boterhoven de Haan, K., Sawyer, M., Ainley, J., & Zubrick, S.R. (2015). *The mental health of children and adolescents: Report on the second Australian child and adolescent survey of mental health and wellbeing*. Commonwealth of Australia. https://www.health.gov.au/resources/publications/the-mental-health-of-children-and-adolescents

[CR34] Longobardi C, Borello L, Thornberg R, Settanni M (2020). Empathy and defending behaviours in school bullying: The mediating role of motivation to defend victims. British Journal of Educational Psychology.

[CR36] Martin AJ, Collie RJ (2019). Teacher-student relationships and students' engagement in high school: Does the number of negative and positive relationships with teachers matter?. Journal of Educational Psychology.

[CR37] Martin AJ, Marsh HW (2008). Academic buoyancy: Towards an understanding of students’ everyday academic resilience. Journal of School Psychology.

[CR38] Mellor D (2005). Normative data for the strengths and difficulties questionnaire in Australia. Australian Psychologist.

[CR39] Memmott-Elison MK, Holmgren HG, Padilla-Walker LM, Hawkins AJ (2020). Associations between prosocial behavior, externalizing behaviors, and internalizing symptoms during adolescence: A meta-analysis. Journal of Adolescence.

[CR40] Metz SM, Frank JL, Reibel D, Cantrell T, Sanders R, Broderick PC (2013). The effectiveness of the learning to BREATHE program on adolescent emotion regulation. Research in Human Development.

[CR42] Muthén, L.K., & Muthén, B. (2017). *Mplus user's guide*. Author.

[CR43] O’Brien, J. (Ed.) (2021). *COVID-19 in Australia.* https://www.covid19data.com.au/about

[CR44] Padilla-Walker LM, Carlo G, Memmott-Elison MK (2018). Longitudinal change in adolescents’ prosocial behavior toward strangers, friends, and family. Journal of Research on Adolescence.

[CR45] Parise M, Canzi E, Olivari MG, Ferrari L (2019). Self-concept clarity and psychological adjustment in adolescence: The mediating role of emotion regulation. Personality and Individual Differences.

[CR46] Quinlan DM, Swain N, Cameron C, Vella-Brodrick DA (2015). How ‘other people matter’ in a classroom-based strengths intervention: Exploring interpersonal strategies and classroom outcomes. The Journal of Positive Psychology.

[CR47] Rodríguez-Meirinhos A, Antolín-Suárez L, Brenning K, Vansteenkiste M, Oliva A (2020). A bright and a dark path to adolescents’ functioning: The role of need satisfaction and need frustration across gender, age, and socioeconomic status. Journal of Happiness Studies.

[CR48] Rose-Krasnor L, Denham S, Rubin KH, Bukowski WM, Laursen B (2009). Social-emotional competence in early childhood. Handbook of peer interactions, relationships, and groups.

[CR49] Roth G, Kanat-Maymon Y, Bibi U (2011). Prevention of school bullying: The important role of autonomy-supportive teaching and internalization of pro-social values. British Journal of Educational Psychology.

[CR50] Ryan RM, Deci EL (2017). Self-determination theory: Basic psychological needs in motivation, development, and wellness.

[CR51] Schroeder, D.A., & Graziano, W.G. (2015). The field of prosocial behavior: An introduction and overview. In D.A. Schroeder & W.G. Graziano (Eds.), *The Oxford handbook of prosocial behavior* (pp.3–34). Oxford University Press, Oxford. 10.1093/oxfordhb/9780195399813.013.32

[CR52] Shrout PE, Bolger N (2002). Mediation in experimental and nonexperimental studies: New procedures and recommendations. Psychological Methods.

[CR53] Skinner EA, Belmont MJ (1993). Motivation in the classroom: Reciprocal effects of teacher behavior and student engagement across the school year. Journal of Educational Psychology.

[CR54] Standage M, Duda JL, Ntoumanis N (2005). A test of self-determination theory in school physical education. British Journal of Educational Psychology.

[CR55] Stump, K.N., Ratliff, J.M., Wu, Y.P., & Hawley, P.H. (2009). Theories of social competence from the top-down to the bottom-up: A case for considering foundational human needs. In J.L. Matson (Ed.), *Social behavior and skills in children* (pp. 23–37). Springer. 10.1007/978-1-4419-0234-4_2

[CR56] Thompson ER (2007). Development and validation of an internationally reliable short-form of the positive and negative affect schedule (PANAS). Journal of Cross-Cultural Psychology.

[CR57] Tomova L, Andrews JL, Blakemore S (2021). The importance of belonging and the avoidance of social risk taking in adolescence. Developmental Review.

[CR58] Weinstein N, Ryan RM (2010). When helping helps: Autonomous motivation for prosocial behavior and its influence on well-being for the helper and recipient. Journal of Personality and Social Psychology.

[CR59] Weissberg RP, Durlak JA, Domitrovich CE, Gullotta TP, Durlak JA, Weissberg RP, Gullotta TP (2015). Social and emotional learning: Past, present, and future. Handbook of social and emotional learning: Research and practice.

[CR60] Wentzel KR, Filisetti L, Looney L (2007). Adolescent prosocial behavior: The role of self-processes and contextual cues. Child Development.

[CR61] Wentzel KR, Muenks K, McNeish D, Russell S (2018). Emotional support, social goals, and classroom behavior: A multilevel, multisite study. Journal of Educational Psychology.

[CR62] White RW (1959). Motivation reconsidered: The concept of competence. Psychological Review.

