# Supplementary material for: Social-emotional need satisfaction, prosocial motivation, and students’ positive behavioral and well-being outcomes
Source: Soc Psychol Educ. 2022 Apr 19;25(2-3):399–424. doi: 10.1007/s11218-022-09691-w (PMC9016699; doi:10.1007/s11218-022-09691-w)
Supplement: Supplementary file 1 — Supplementary file1 (DOCX 20 kb) [file 11218_2022_9691_MOESM1_ESM.docx]

**Online Supplementary Materials for**

**Social-Emotional Need Satisfaction, Prosocial Motivation, and Students’ Positive Behavioral and Well-Being Outcomes**

**Measurement Invariance**

Multigroup CFA was employed to test measurement invariance of the substantive latent factors in the model: social-emotional need satisfaction, prosocial motivation, and emotional well-being. Population subgroups across three background characteristics were considered: gender (male vs. female), age (median split), and socio-economic status (median split). Measurement invariance was tested with three models for each of the background characteristics: configural model (all parameters free across subgroups), metric (loadings constrained across subgroups), and scalar (loadings and intercepts constrained across subgroups). Changes in fit indices within cut-offs (i.e., ΔRMSEA ≤ .015 and ΔCFI/TLI of ≤ -.01) indicate evidence of invariance (Chen, 2007; Cheung & Rensvold, 2002). As such, it is possible to conclude that the loadings and intercepts were equivalent across the subgroups examined. Table S1 shows fit indices from these tests and demonstrates that measurement invariance was obtained.

**Results Involving Background Characteristics**

The structural equation model (see Table 3 in main manuscript) demonstrated that female students exhibited significantly higher prosocial behavior, but also reported greater negative affect. Older students reported lower autonomous motivation. Having a non-English speaking language background was associated with greater perceived autonomy. Being a student with ADHD was associated with lower perceived social competence, and lower perceived relatedness with both students and teachers, greater conduct problems, and greater negative affect.

Three notable findings involving covariates are considered here. Compared to students without ADHD, students with ADHD reported lower perceived social competence and lower relatedness (with both students and teachers). Students with ADHD are known to experience difficulties with impulse control and conflict resolution (e.g., Johnson et al., 2008), which may explain the lower levels of perceived social competence. Research has also shown that students with ADHD often report lower perceived relatedness compared to students without ADHD (Martin et al., 2017). Given the importance of need satisfaction for positive individual functioning in general, future research that examines how these factors can be boosted among students with ADHD is crucial.

Table S1

*Measurement Invariance Tests*

|  | RMSEA | CFI | TLI |
| --- | --- | --- | --- |
| *Gender* |  |  |  |
| Configural | .045 | .922 | .913 |
| Metric | .044 | .922 | .916 |
| Scalar | .046 | .915 | .910 |
| *Age* |  |  |  |
| Configural | .042 | .930 | .922 |
| Metric | .042 | .929 | .923 |
| Scalar | .041 | .928 | .924 |
| *Socio-economic status* |  |  |  |
| Configural | .043 | .926 | .918 |
| Metric | .043 | .924 | .918 |
| Scalar | .043 | .923 | .918 |

**References**

Chen, F.F. (2007). Sensitivity of goodness of fit indices to lack of measurement invariance. *Structural Equation Modeling: A Multidisciplinary Journal, 14*(3), 464-504. http://dx.doi.org/10.1080/10705510701301834

Cheung, G.W., & Rensvold, R.B. (2002). Evaluating goodness-of-fit indexes for testing measurement invariance. *Structural Equation Modeling: A Multidisciplinary Journal, 9(*2), 233-255. http://dx.doi.org/10.1207/S15328007SEM0902_5

Johnson, K. A., Robertson, I. H., Barry, E., Mulligan, A., Dáibhis, A., Daly, M., Watchorn, A., Gill, M., & Bellgrove, M. A. (2008). Impaired conflict resolution and alerting in children with ADHD: Evidence from the Attention Network Task (ANT). *Journal of Child Psychology and Psychiatry, 49*(12), 1339–1347. https://doi.org/10.1111/j.1469-7610.2008.01936.x

Martin, A.J., Burns, E.C., & Collie, R.J. (2017). ADHD, personal and interpersonal agency, and achievement: Exploring a proposed process based on social cognitive theory. *Contemporary Educational Psychology, 50*, 13-22. https://dx.doi.org/10.1016/j.cedpsych.2016.12.001

Shrout, P.E., & Bolger, N. (2002). Mediation in experimental and nonexperimental studies: New procedures and recommendations. *Psychological Methods, 7*, 422-445. http://dx.doi.org/10.1037/1082-989X.7.4.422
